# Supplementary material for: From sequence to enzyme mechanism using multi-label machine learning
Source: BMC Bioinformatics. 2014 May 19;15:150. doi: 10.1186/1471-2105-15-150 (PMC4229970; doi:10.1186/1471-2105-15-150)
Supplement: Additional file 2 — Java code of ml2db. Additional file ml2db_code.tar.gz contains the Java source code to run the multi-label machine learning experiments and save the results to database. The code’s Javadoc is included. [file 1471-2105-15-150-S2.zip › additional file 2/ml2db/ml2db/doc/mulan/data/LabelNode.html]

LabelNode


JavaScript is disabled on your browser.


- Overview
- Package
- Class
- Use
- Tree
- Deprecated
- Index
- Help

- Prev Class
- Next Class

- Frames
- No Frames

- All Classes

- Summary:
- Nested |
- Field |
- Constr |
- Method

- Detail:
- Field |
- Constr |
- Method


mulan.data

## Interface LabelNode

- All Known Implementing Classes:
  :   LabelNodeImpl

  ---

    

  ```
  public interface LabelNode
  ```

  Represents a label attribute as a node in the labels hierarchy.
  The identity of the node is represented by label name, which must be unique
  within labels hierarchy.

  Author:
  :   Jozef Vilcek

- - ### Method Summary

    Methods

    | Modifier and Type | Method and Description |
    | --- | --- |
    | `java.util.Set<LabelNode>` | `getChildren()` Gets the unmodifiable `Set` of child `LabelNode` of this node, if hierarchy exists. |
    | `java.util.Set<java.lang.String>` | `getDescendantLabels()` Gets a `Set` of the names of descendant `LabelNode` of this node, if hierarchy exists. |
    | `java.lang.String` | `getName()` Gets the name of the label this node represents. |
    | `LabelNode` | `getParent()` Gets the parent `LabelNode` of this node if hierarchy exists. |
    | `boolean` | `hasChildren()` Determines whether the `LabelNode` has child nodes. |
    | `boolean` | `hasParent()` Determines whether the `LabelNode` has a parent node in a hierarchy. |

- - ### Method Detail


    - #### getName

      ```
      java.lang.String getName()
      ```

      Gets the name of the label this node represents.
      The name corresponds to the ID of label attribute in the arff data set.
      The name of the label must be unique within the data set, because the
      identity of `LabelNode` is determined by the name.

      Returns:
      :   the name of the label this node represents


    - #### hasParent

      ```
      boolean hasParent()
      ```

      Determines whether the `LabelNode` has a parent node in a hierarchy.

      Returns:
      :   `true` if the node has parent; `false` otherwise.


    - #### getParent

      ```
      LabelNode getParent()
      ```

      Gets the parent `LabelNode` of this node if hierarchy exists.
      If the node has not a parent `LabelNode`, `null` is returned.

      Returns:
      :   the parent `LabelNode` or `null` if the parent does not exists.


    - #### hasChildren

      ```
      boolean hasChildren()
      ```

      Determines whether the `LabelNode` has child nodes.

      Returns:
      :   `true` if the node has child nodes; `false` otherwise.


    - #### getChildren

      ```
      java.util.Set<LabelNode> getChildren()
      ```

      Gets the unmodifiable `Set` of child `LabelNode` of this node, if hierarchy exists.
      If no child nodes exists for this `LabelNode`, empty `Set` is returned.

      Returns:
      :   the `Set` of child nodes


    - #### getDescendantLabels

      ```
      java.util.Set<java.lang.String> getDescendantLabels()
      ```

      Gets a `Set` of the names of descendant `LabelNode` of this node, if hierarchy exists.
      If no descendant nodes exists for this `LabelNode`, empty `Set` is returned.

      Returns:
      :   the `Set` of child nodes


- Overview
- Package
- Class
- Use
- Tree
- Deprecated
- Index
- Help

- Prev Class
- Next Class

- Frames
- No Frames

- All Classes

- Summary:
- Nested |
- Field |
- Constr |
- Method

- Detail:
- Field |
- Constr |
- Method
